# Genetic Variants and Related Biomarkers in Sporadic Alzheimer’s Disease

**DOI:** 10.1007/s40142-014-0062-6

**Published:** 2014-12-24

**Authors:** Rita Guerreiro, Jose Bras, Jamie Toombs, Amanda Heslegrave, John Hardy, Henrik Zetterberg

**Affiliations:** 1 Department of Molecular Neuroscience, UCL Institute of Neurology, Queen Square, London, WC1N 3BG UK; 2 Clinical Neurochemistry Laboratory, Department of Psychiatry and Neurochemistry, Institute of Neuroscience and Physiology, The Sahlgrenska Academy at University of Gothenburg, Mölndal, Sweden; 3 Department of Molecular Neuroscience, UCL Institute of Neurology, 1 Wakefield Street (1st Floor), London, WC1N 1PJ UK

**Keywords:** Cerebrospinal fluid, Imaging biomarkers, Novel susceptibility genes, Risk alleles

## Abstract

From a neuropathological perspective, elderly patients who die with a clinical diagnosis of sporadic Alzheimer’s disease (AD) are a heterogeneous group with several different pathologies contributing to the AD phenotype. This poses a challenge when searching for low effect size susceptibility genes for AD. Further, control groups may be contaminated by significant numbers of preclinical AD patients, which also reduces the power of genetic association studies. Here, we discuss how cerebrospinal fluid and imaging biomarkers can be used to increase the chance of finding novel susceptibility genes and as a means to study the functional consequences of risk alleles.

## Introduction

Alzheimer’s disease (AD) is considered the most common form of dementia, but still lacks effective preventive or therapeutic interventions. This is probably partially due to an incomplete understanding of AD aetiology and the possible confounding factors associated with its genotypic and phenotypic heterogeneity. The disease was named after Alois Alzheimer, who in the early 20th century described cases of “presenile dementia”, with neuropathological features characterized by gross cerebral atrophy, extracellular senile plaques and intracellular neurofibrillary tangles. Although there were some early doubts about the distinction between early-onset and late-onset dementia, during the first half of the 20th century, AD was mainly thought of as a rare disease that affected middle-aged people, while most elderly people with dementia were considered to have “senile dementia”, caused primarily by age-related vascular pathology [1]. AD was only recognized as an important cause of dementia in elderly people after several autopsy studies in the 1950s to 1970s had noted the high prevalence of AD-like neuropathology in patients with “senile dementia” [2, 3].

During the 1980s to 1990s, breakthroughs in biochemistry and genetics laid the basis for strong hypotheses about the cause of AD, which first led to the development of the symptomatic treatments that are currently available [4], and second to clinical trials of therapeutic approaches targeted against amyloid β (Aβ), the major component of senile plaques and a potential driver of the disease [5, 6].

Several lines of data point to significant pathological and clinical heterogeneity among clinically diagnosed AD patients. Many autopsy studies have shown that most elderly patients with dementia have mixed pathologies, with AD-like pathology combined with other brain pathologies, such as Lewy bodies, white matter disease, angiopathy, or TDP-43 inclusions [7–10]. After AD, the most common dementia form is dementia with Lewy bodies (DLB), characterized by the accumulation of α-synuclein aggregates and cognitive impairment that is not dominated by memory decline, but rather executive and visuospatial problems, and a high frequency of hallucinations and delusions. About 10–40 % of AD patients have concomitant Lewy bodies [11–13], which likely affects the clinical course of AD, since AD patients with Lewy bodies have faster cognitive decline than those without Lewy bodies [14]. So far, it remains very difficult to identify Lewy body pathology in AD patients in vivo. Another very common cause of dementia is cerebrovascular disease. Vascular cognitive impairment defines alterations in cognition, ranging from subtle deficits to full-blown dementia, attributable to cerebrovascular causes. Often coexisting with AD, mixed vascular and neurodegenerative dementia has actually been proposed as the leading cause of age-related cognitive impairment and dementia [15]. Biomarker changes that associate with cerebrovascular disease, e.g., white matter changes on computed tomography or magnetic resonance imaging (MRI) of the brain and elevated CSF levels of neurofilament light, are common in elderly patients with clinical AD [16], and it is possible that the dementia syndrome in some of these individuals is not driven by AD pathology but rather deficits in the cerebrovasculature. Susceptibility genes for AD pathology will not be found in these patients.

Here, we share our view on what could be gained by performing genetic studies on patients with more extensive information on underlying pathologies using different forms of biomarkers. The focus is on AD-related pathologies but the reasoning should be relevant also to other neurodegenerative diseases.

## Biomarkers for AD Pathology

During the last two decades, biomarker tools have been developed, which allow researchers and clinicians to identify AD-like pathology in vivo, even years before the first symptoms emerge. Cerebrospinal fluid (CSF) levels of total tau and phospho-tau are positively correlated to neurodegeneration and neurofibrillary tangle pathology, whereas CSF levels of aggregation-prone 42 amino acid long Aβ (Aβ42) are negatively correlated to plaque pathology [17]. A recent meta-analysis assessing studies in which clinical criteria were used suggests that the combination of CSF tau and Aβ markers shows a sensitivity of 84 % (76–90 %) and a specificity of 71 % (59–81 %) for AD both in dementia and mild cognitive impairment stages of the disease [18]. Further, plaque pathology can be visualized using amyloid positron emission tomography (PET) [19], and tau PET is a more recent potential biomarker tool to monitor tangle pathology [20]. The first biomarker changes indicating Aβ build-up in the brain appear 10–20 years before clinical onset of the disease with Aβ markers preceding tau markers by 5–10 years [21, 22•]. This puts Aβ before tau in regards to the sequence of events during the disease process. However, much remains to be learnt regarding what factors may initiate Aβ deposition.

## Genetics of Sporadic AD

### APP and PSEN Mutations

The causative roles of mutations in the *amyloid β precursor protein* (*APP*) and *presenilin* (*PSEN1* and *PSEN2*; encoding the active site of γ-secretase that produces Aβ from APP with most mutations resulting in qualitative changes in APP-processing which promote cerebral β-amyloidosis [23]) genes in familial AD have long been recognized [24]. However, genetic analysis of late-onset sporadic AD has surprisingly revealed that these mutations are also pathogenic in some cases of late-onset AD and CSF biomarkers have been used as endophenotypes to detect mutations in the genes known to harbour AD-causative mutations [25, 26]. The Swedish *APP* mutation causes AD because it makes the protein a better substrate for BACE1 (the major β-secretase responsible for cleaving APP in the N-terminal part of the Aβ domain making the remaining stub a γ-secretase substrate) and thus more APP is metabolized along the amyloidogenic pathway and more Aβ is produced [27]. In a recent study, Jonsson and colleagues [28•] noted that a specific mutation in *APP*, which previously had been identified to be located close to the β-secretase site [29], made it a worse substrate for BACE1 and correspondingly was associated with lower Aβ production and lower risk of AD. This observation, if replicated, supports the Aβ cascade hypothesis and also BACE1 inhibition as a valid target for AD therapy. Autosomal dominant mutations that cause familial AD without effect on Aβ metabolism have not yet been reported. The study of AD biomarkers in these familial cases has been essential to establish the timeline of pathological events in the disease, and in particular, to support the existence of a long preclinical stage [22•, 30].

A recent multicenter, longitudinal study of CSF in families with autosomal dominant AD mutations revealed a clear transition of CSF markers over-time with reduced concentrations of CSF Aβ1-42 (associated with the presence of amyloid plaques) and elevated concentrations of CSF markers of neurofibrillary tangles and neuronal injury/death in asymptomatic mutation carriers 10–20 years before their estimated age at symptom onset. The longitudinal assessment also revealed an over-time decrease in the concentration of injury-related markers after symptom onset, suggesting a slowing of acute neurodegenerative processes with symptomatic disease progression [22•].

### APOE

The association of the *apolipoprotein E* (*APOE*) ε4 allele with AD is strong (odds ratios ranging from 3 to 10 in different studies [31•]) and undisputed. ApoE is the major carrier of cholesterol in the CNS and has also important roles in Aβ metabolism, aggregation, and deposition. Increased plaque deposition has been observed in *APOE* ε4-positive individuals and in *APOE* ε4 knock-in animal models of cerebral β-amyloidosis [32–34]. ApoE binds Aβ but the apoE4 isoform has a lower affinity than apoE3, and it appears that at least part of the association of *APOE* ε4 with Aβ plaque pathology is related to apoE4 being less efficient in clearing Aβ from the brain parenchyma [35]. This may explain why cognitively healthy people with *APOE* ε4 have biomarker signs of Aβ pathology at an earlier age than people lacking the *APOE* ε4 allele (especially compared to people carrying the *APOE* ε2 allele) [36].

In a genome-wide study, the *APOE* ε4 genotype was the strongest single-genetic factor associated with CSF ApoE protein levels. ApoE CSF, but not plasma, levels were found to significantly associate with CSF Aβ42 levels independently of the *APOE* ε4 genotype, and suggesting that ApoE levels in CSF may be a useful endophenotype for AD [37]. However, in contrast, *APOE* ε4 does not interact with age to produce biomarker signs of axonal degeneration (increased CSF T-tau) or tangle pathology (increased CSF P-tau), supporting the view that *APOE* ε4 does not have a primary effect on these aspects of AD pathology (alternatively, these biomarkers may have too low sensitivity to identify such effects).

One study [38] has shown an interesting interaction effect between *APOE* and Aβ1-42 in the CSF of *APOE* ε4 carriers. Homo- and heterozygotes of the *APOE* ε4 allele had significantly lower detectable Aβ42 concentrations than *APOE* ε3 homozygotes. Although the exact mechanism is not understood, the implication is for matrix composition of these (and potentially other) proteins in CSF to impact the measurement of corner-stone biomarkers such as Aβ42, and perhaps aid exploration of patho-relevant physiological processes. As such there may be considerable utility for genetic and proteomic characterisation of AD patients and research subjects.

Exactly how the ApoE4 isoform promotes AD is still unclear, and conflicting results present in the literature support both a loss of positive or gain of negative functions of the protein. The role of ApoE in AD becomes even more complex when considering the recent report of a patient with a rare form of severe dysbetalipoproteinemia who was homozygous for an ablative *APOE* frameshift mutation. As expected, the patient had exceptionally high cholesterol content with profound lipoprotein metabolism dysregulation. However, this 40-year-old patient presented surprisingly normal neurological-related features (normal vision, normal cognitive, neurological, and retinal functions, normal findings on brain magnetic resonance imaging, and normal CSF levels of Aβ and tau proteins) [39]. It would have been very interesting to determine the CSF lipidation profile of this patient, especially to assess the possibility of compensation by other apolipoproteins, and further follow-up will reveal if age-related neurological deficits will appear.

### Other Susceptibility Genes

Genome-wide association studies (GWAS) identify common loci (typically frequencies of 10–50 %), which have low to modest effects on risk (typically with odds ratios in the 1.1–2.0 range). Over the last 5 years, this approach has begun to yield large numbers of risk loci, and this harvest continues as study pooling is ongoing and as larger numbers of samples are collected [40••]. The utility of these studies in terms of predicting who will develop disease is currently modest. However, their larger importance is that they may identify pathways and processes in which genetic variability affects disease risk. So far, GWAS have identified 3 such pathways: (i) endosomal vesicle recycling (*BIN1*, *PICALM* and *SORL1*), (ii) the innate immune system (*TREM2*, *CR1* and *CLU*) and (iii) genes related to cholesterol metabolism (*ABCA7*, *CLU*) [41]. It is not yet possible to definitively relate these pathways directly to each other or to Aβ but they resonate well with recent CSF biomarker data showing links between Aβ pathology and/or AD and CSF levels of endosomal/lysosomal network proteins, and proteins related to microglial activation and synaptic function or integrity [42–45].

## Can Biomarkers Help us Finding More Risk Genes for Sporadic AD?

There has been a recent surge in interest in the use of endophenotypes in research on psychiatric and neurodegenerative diseases, AD in particular. The concept was introduced by Gottesman and Shields to reduce the harmful influence of poor accuracy in the clinical diagnosis of psychiatric and neurological diseases on the power of genetic association studies [46], which is a major problem in AD research. In addition, the identification of disease endophenotypes offers the prospect of creating experimental models relevant to human pathophysiology, which will be suitable for experimental approaches and greatly facilitate the development and screening of novel therapeutics. Endophenotypes may be described as internal phenotypes that lie on the pathway between genes and disease. Fundamental to the concept is the assumption that variation in an endophenotype will depend upon variation in fewer genes than the more complex disease phenotype and therefore be more tractable to genetic analysis [46]. The combination of clinical and biomarker information (as opposed to definition based on clinical data only) to define cases and controls in genetic association studies increases the power of these analyses. This could be inferred when *APOE* ε4 was found to present a stronger association with AD when clinical criteria incorporated biomarker information, and when genetic associations were replicated using much smaller sample sizes when compared to the original associations, by means of defining cases and controls according to CSF biomarker profiles [31•].

**Fig. 1:**
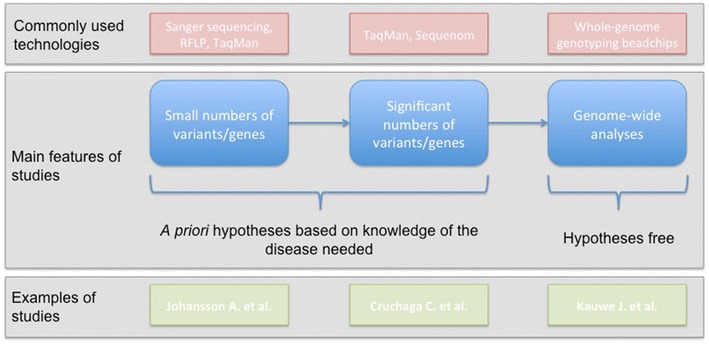
Evolution of genetic studies based on endophenotype associations in Alzheimer’s disease. Examples of studies [47, 48•, 49]

In AD, CSF and imaging biomarkers have been used as endophenotypes in several genetic studies, both to increase the chance of finding novel susceptibility genes and as a means to study the functional consequences of risk alleles. These studies have been closely tied with genetic technology developments, moving from analyses of individual genetic variants or genes to genome-wide approaches (Fig. 1).

When studying specific variants/genes, these were usually chosen given an a priori biological or aetiological association with disease. This was the case when Kauwe et al. identified a gene-physiological environment interaction between *MAPT* common single-nucleotide polymorphisms (SNPs) and Aβ deposition through the evaluation of the role of these SNPs in CSF tau/ptau levels [50]. An analogous approach was used to study genes involved in the complement system. Daborg et al. chose to study the complement system because of its involvement in both physiological and AD synapse elimination. The authors studied 4 SNPs in different genes (*C2*, *C3*, *CFB* and *CR1*) and although no significant associations were found with AD risk, potential associations between SNPs in *C2* and *CFB* were identified in relation to CSF tau levels and Mini-Mental State Examination (MMSE) scores [51].

Cruchaga and colleagues studied 384 SNPs selected from genes known to code for the most relevant tau kinases, phosphatases, and in other genes implicated in other posttranslational modifications of tau, or tau degradation. The authors were able to detect a SNP (rs1868402) in *PPP3R1* associated with CSF P-tau181 levels. This variant showed a strong association with the rate of decline in AD patients, but no association was detected with AD risk or age at onset of the disease [49]. By taking a genome-wide approach, the same group was able to identify 4 genome-wide significant signals associated with CSF tau levels (including tau and ptau): *APOE*; rs9877502 located at 3q28 between *GEMC1* and *OSTN*; rs514716 located at 9p24.2 within *GLIS3*; and rs6922617 at 6p21.1 within the *TREM* gene cluster [52•]. A clear signal that this is a valid approach to identify novel risk variants for a complex disease like Alzheimer’s is the fact that 3 (*APOE*, 3q28 and 6p21.1) of the 4 genome-wide significant loci identified had also been independently associated with the disease [53, 54, 55••, 56••].

To expand the use of endophenotypes beyond CSF Αβ42 and tau, Kauwe et al. studied the CSF levels of 59 AD-related proteins in a GWAS study [48•]. They identified significant genetic associations with CSF levels of 5 proteins involved in amyloid processing and pro-inflammatory signalling: Angiotensin-converting enzyme (ACE), Chemokine (C–C motif) ligand 2 (CCL2), Chemokine (C–C motif) ligand 4 (CCL4), Interleukin 6 receptor (IL6R) and Matrix metalloproteinase-3 (MMP3), suggesting mechanisms for genetic control of CSF and plasma levels of these disease-related proteins. Interestingly, the SNPs found to be significantly associated in *ACE* and *MMP3* also showed association with AD risk [48•].

## Can Biomarkers Help us to Characterize the Functional Mechanisms of each Associated Loci in AD?

One of the limitations of case–control GWAS is the fact that generally only haplotype tagging markers are identified by this methodology. When comparing frequencies of genotypes between large numbers of cases and controls, it would be very difficult to do this for every single variant in the genome. To overcome this, GWAS platforms are based on haplotypes and only SNPs tagging each haplotype are analysed. Although this approach eases the burden of comparative testing, it also prevents the identification of specific disease-associated variants, as these can be in linkage disequilibrium (LD) with the genome-wide hit identified. An essential follow-up step to the identification of GWAS significant loci for a disease is the characterization of the functional mechanisms by which the associated variants influence the risk for disease. Kauwe et al. used an endophenotype-based approach to attempt to generate biological hypotheses of risk mechanism for *BIN1*, *CLU*, *CR1* and *PICALM*. To accomplish this, the authors sampled common variation in these genes, genotyping 355 variants in over 600 individuals for whom measurements of CSF Aβ42 and P-tau181 had been obtained. Although this was a well-designed study, no associations between SNPs in these genes and CSF Aβ42 or P-tau181 levels were found in the studied sample, suggesting that the associated variants at these loci do not affect risk via a mechanism resulting in a strong additive effect on CSF levels of Aβ42 or P-tau181 [57]. In a study using family-based and case–control designs, Schjeide et al. performed an analogous analysis of 5 variants in *CLU*, *CR1* and *PICALM*. The authors identified a significant effect of rs541458 in *PICALM* on CSF Aβ42 levels [58]. With the same goal, Elias-Sonnenschein et al. studied 36 SNPs in 25AD-related genes in a cohort of 222 Finish AD patients for which CSF biomarker levels were available. They identified several significant associations: *APOE* ε4, *CLU* rs11136000, and *MS4A4A* rs2304933 correlated with significantly decreased CSF Aβ42; at an uncorrected level *PPP3R1* rs1868402 and *MAPT* rs2435211 were related with increased T-tau; *SORL1* rs73595277 and *MAPT* rs16940758 were associated with increased P-tau [59].

Altogether, these studies clearly point to the need of structured, well-powered analyses. The application of this approach to other loci, the increase in the number of samples studied and the use of replication cohorts will probably allow for a deeper characterization of these associations.

## Conclusions

The integration of genetic results with biomarkers is essential for advancing the research into AD and other complex disorders. Genetic studies clearly have an extreme potential for the identification of novel biomarkers for AD, but biomarkers are also essential for the guiding of genetic studies both in familial and sporadic forms of the disease. The use of endophenotypes in GWAS adds a layer of information to this type of study because it directly associates with specific disease-related biological mechanisms. The computational ability to test for associations in well-structured studies at a genome-proteome-wide level will most likely reveal novel molecular interactions important for the risk and progression of AD.
